# Removal of Incarcerated Screws with Damaged Heads using a Novel Technique of Creating Two Straight and Parallel Planes on the Screw Head with a High Speed Diamond-Tipped Burr and Stable Grip of a Locking Plier

**DOI:** 10.5704/MOJ.2111.026

**Published:** 2021-11

**Authors:** B Hii, MR Draman, S Singh

**Affiliations:** Department of Orthopaedic Surgery (NOCERAL), University of Malaya, Kuala Lumpur, Malaysia

Dear editors,

Incarcerated screws pose a challenging task to many junior orthopaedic surgeons who hone their skills through experience. One must first have sufficient knowledge regarding orthopaedic implants and the methods of their removal, and should have at their disposal a variety of techniques should one come across difficulty intra-operatively. We recently encountered a case of removal of femoral nail which was complicated with incarcerated screws. The screws were eventually removed using a technique of creating two straight and parallel planes on the screw head with a high speed diamond-tipped burr and stable grip of a locking plier.

**Fig 1:**
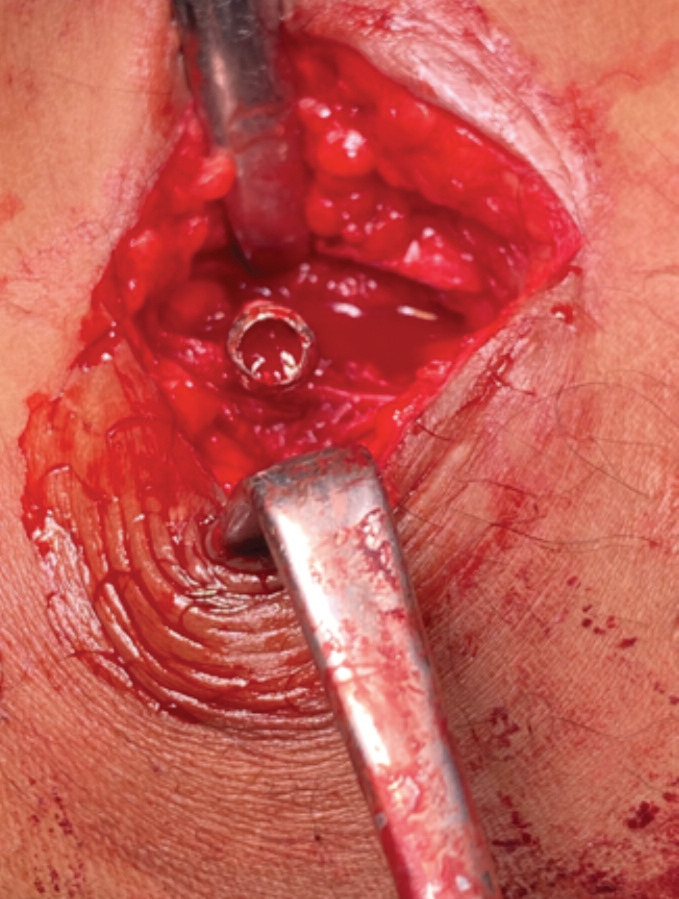
Damaged hexagonal recess of the screw head.

The patient was a 26-year-old gentleman who was referred to our trauma unit for removal of implant in view of left knee instability with suspicion of left posterior cruciate ligament injury requiring confirmation of diagnosis by magnetic resonance imaging (MRI). He had a history of left femur fracture in which a titanium retrograde nail was inserted four years ago which had achieved union. During the surgery, the most proximal locking screw and distal two locking screws were found to be well integrated with bony overgrowth over the screw heads. Upon clearing the bone within and around the screw heads, attempt at removal of the screws using the conventional way failed and repeated attempts led to stripped screws (Fig. 1).

**Fig 2:**
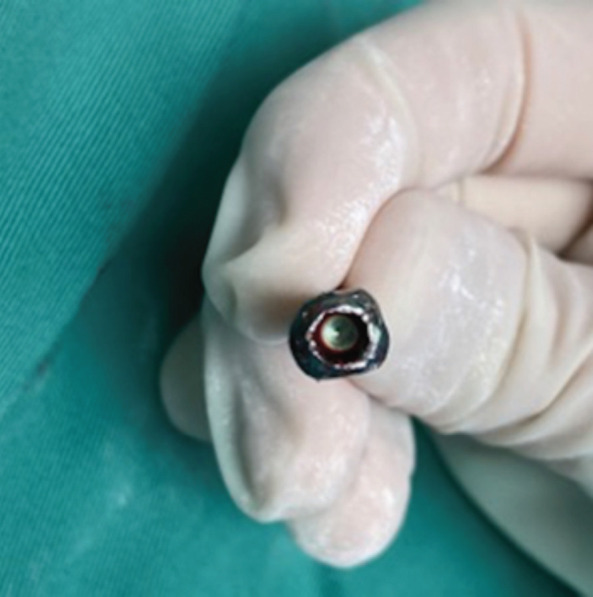
Two straight and parallel planes formed on the screw head using the high speed diamond-tipped burr.

**Fig 3:**
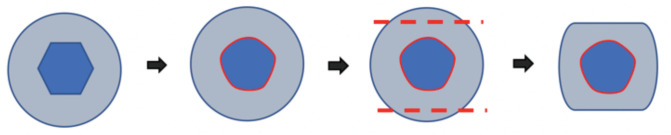
Diagram depicting a screw head that becomes damaged at the hexagonal recess; dotted lines show the straight and parallel planes created using a high speed diamond-tipped burr.

Further attempts using the conical extraction screw from the universal screw removal set were futile as it did not engage within the screw heads. We believe this was because the screw was tightly jammed due to osteointegration^1^. We also attempted to grasp the screw heads with locking plier, but failed due to the round shape of the screw heads. To improve the grip of the locking plier, the surgeon proceeded to form two straight and parallel planes on the screw head by using a high speed diamond-tipped burr (Fig. 2). The flattening of the rounded surface on the screws allowed a stable grip for the pliers and the screws were finally removed using gentle anticlockwise and pulling force. A diagram depicting a stripped screws, where straight and parallel planes are created using high speed diamond-tipped burr is shown (Fig. 3).

Incarcerated screws pose a challenging and delicate situation in removal of an implant^2^. The procedure should never be underestimated and thorough pre-operative planning with regards to types of implants and removal instruments should be prepared. Surgeons should equip themselves with broad knowledge and should have at their disposal various methods of removal of implant should they come across any difficulties intra-operatively. The above technique would be a handy method to be added into a trauma surgeon’s armamentarium in removing incarcerated screws.
